# Alteration of Liver Enzymes Is a Feature of the *Myh9*-Related Disease Syndrome

**DOI:** 10.1371/journal.pone.0035986

**Published:** 2012-04-25

**Authors:** Alessandro Pecci, Ginevra Biino, Tiziana Fierro, Valeria Bozzi, Annamaria Mezzasoma, Patrizia Noris, Ugo Ramenghi, Giuseppe Loffredo, Fabrizio Fabris, Stefania Momi, Umberto Magrini, Mario Pirastu, Anna Savoia, Carlo Balduini, Paolo Gresele, for the Italian Registry for MYH9-releated diseases

**Affiliations:** 1 Department of Internal Medicine, IRCCS Policlinico San Matteo Foundation and University of Pavia, Pavia, Italy; 2 Institute of Molecular Genetics, CNR, Pavia, Italy; 3 Division of Internal and Cardiovascular Medicine, Department of Internal Medicine, University of Perugia, Perugia, Italy; 4 Department of Pediatrics, University of Torino, Torino, Italy; 5 Department of Oncology, Azienda “Santobono-Pausilipon", Pausilipon Hospital, Napoli, Italy; 6 Department of Medical Sciences, University of Padova, Padova, Italy; 7 Department of Pathology, University of Pavia, Pavia, Italy; 8 Institution of Population Genetics, CNR, Sassari, Italy; 9 Laboratory of Genetics, Institute for Maternal and Child Health – IRCCS “Burlo Garofolo", Trieste, Italy; Heart Center Munich, Germany

## Abstract

**Background:**

*MYH9*-related disease (*MYH9*-RD) is a rare autosomal dominant genetic syndrome characterized by congenital thrombocytopenia associated with the risk of developing progressive nephropathy, sensorineural deafness, and presenile cataract. During the collection of a large case-series of patients with *MYH9*-RD we noticed several cases with unexplained elevation of liver enzymes. Our aim was to evaluate if the alteration of liver tests is a feature of the *MYH9*-RD and to define its clinical significance.

**Methods and Findings:**

Data concerning liver tests, prospectively recorded in the Italian Registry for *MYH9*-RD, were collected and compared with those of three control populations: patients with autoimmune thrombocytopenia, patients with inherited thrombocytopenias other than *MYH9*-RD, and the participants to a large epidemiologic survey in an Italian geographic isolate. Thirty-eight of 75 evaluable *MYH9-RD* patients (50.7%) showed an elevation of ALT and/or AST, and 17 of 63 (27.0%) an increase of GGT. The increases ranged from 1.9±0.7 to 2.7±1.6 fold the upper normal limit. The prevalence of liver test alterations was significantly higher in *MYH9-RD* patients than in each of the control populations, with odds ratios ranging from 8.2 (95% CIs 2.2–44.8) to 24.7 (14.8–40.8). Clinical follow-up and more detailed liver studies of a subset of patients, including ultrasound liver scan, liver elastography and liver biopsy in one case, did not show any significant structural damage or evolution towards liver insufficiency.

**Conclusions:**

Elevation of liver enzymes is a frequent and previously unrecognized feature of the *MYH9*-RD syndrome; however, this defect does not appear to have poor prognostic value.

## Introduction

*MYH9*-related disease (*MYH9*-RD) is an autosomal dominant disorder characterized by congenital thrombocytopenia with giant platelets associated with the risk of developing progressive nephropathy during infancy or adult life, sensorineural deafness and presenile cataract [1], [2]. *MYH9*-RD encompasses a series of autosomal dominant macrothrombocytopenias previously considered as distinct disorders, namely May-Hegglin anomaly (MHA; MIM#155100), Sebastian Syndrome (SBS; MIM#605249), Fechtner Syndrome (FTNS; MIM#153640), and Epstein Syndrome (EPTS; MIM#135650), all deriving from mutations in the *MYH9* gene encoding for the heavy chain of nonmuscle myosin-IIA (NMMHC-IIA) [1]–[5]. At least 44 different *MYH9* mutations have been reported so far [1], [3]–[9], which may involve either the N-terminal motor domain (MD) or the C-terminal tail domain (TD) of NMMHC-IIA. Genotype-phenotype correlation studies have shown that patients with mutations affecting the MD have more severe thrombocytopenia and higher incidence of nephropathy and deafness than those with mutations involving the TD [2]. Myosin-IIA is a non-sarcomeric myosin expressed in most cells and tissues where it participates in functions associated with the generation of chemomechanical forces by the cytoskeleton, including cytokinesis, cell motility and maintenance of cell shape [10], [11]. Studies on the murine orthologue *Myh9* have shown that it is expressed in liver, kidney, lung and spleen and, at a lower level, in heart and brain, with no expression in skeletal muscle and testis [12]. Although congenital macrothrombocytopenia, sensorineural hearing loss, nephropathy and cataract are the only recognized clinical hallmarks of the *MYH9*-RD syndrome, previous studies have reported altered liver function tests in single cases or small case series of patients with *MYH9*-RD [13]–[15]. During the collection of a large database of *MYH9*-RD patients (Italian Registry for *MYH9*-RD, www.registromyh9.org) we noticed several cases with unexplained elevated liver enzymes. Aims of the present study were to carry out a systematic evaluation of the prevalence of alterations of liver enzymes in a large, well-characterized *MYH9*-RD population, to compare it with the prevalence observed in a large population survey performed in central eastern Sardinia [16] and in two populations of patients with thrombocytopenias not related to *MYH9* mutations studied during the same period at the authors' Centers, and to assess the evolution of the liver test alterations in terms of potential organ damage.

## Methods

### Patients

The study included all the consecutive patients enrolled in the Italian Registry for *MYH9*-RD for which data concerning liver function were available. Diagnosis of *MYH9*-RD was confirmed in all patients by both immunofluorescence screening test and molecular analysis [1], [2], [17]. Most patients have been already reported (Table 1). The study was approved by the Ethic Committee of the IRCCS Policlinico San Matteo Foundation, Pavia; written informed consent was obtained from all the patients or their legal guardians. As a control, data from the participants to the Ogliastra cross-sectional epidemiologic survey, carried out in central eastern Sardinia between with 2002 and 2008, were used [16], [18]. In addition, data of liver enzymes of *MYH9*-RD patients were compared with those of two populations of consecutive patients with *MYH9*-RD-unrelated thrombocytopenias studied during the same period at the authors' institutions, for which we had single or repeated liver enzyme determinations: one with acquired immune thrombocytopenia (ITP), the other with different forms of inherited thrombocytopenia [19].

**Table 1:** Genetic analysis of the 75 *MYH9*-RD patients described in this study.

Aminoacid change	Nucleotide change	n° of patients (n° of families)		
		Enrolled	Previously reported	Ref.
p.A95D	c.284C>A	2 (1)	2 (1)	9
p.S96L	c.287C>T	2 (2)	1 (1)	9
p.R702C	c.2104C>T	11 (10)	9 (9)	9
p.R702H	c.2105G>A	2 (2)	2 (2)	9
p.E1066-A1072del	c.3195_3215del	1 (1)	1(1)	9
p.E1066-A1072dup	c.3195_3215dup	1 (1)	1(1)	9
p.T1155I	c.3464C>T	1 (1)	1 (1)	9
p.T1155A	c.3463A>G	1 (1)	1 (1)	9
p.R1162T	c.3485G>C	2 (2)	2 (2)	33
p.R1165C	3493C>T	4 (4)	3 (3)	9
p.R1165L	c.3494G>T	2 (2)	1 (1)	9
p.D1424H	c.4270G>C	7 (4)	7 (4)	9,34
p.D1424N	c.4270G>A	2 (1)	2 (1)	9
p.D1424Y	c.4270G>T	1 (1)	none	-
p.D1447V	c.4340A>T	5 (2)	4 (1)	9
p.E1841K	c.5521G>A	6 (3)	6 (3)	9
p.G1924RfsX21	c.5770_5779del	1 (1)	1 (1)	9
p.D1925TfsX23	c.5770delG	3 (1)	3 (1)	9
p.V1930CfsX18	c.5788delG	1 (1)	1 (1)	9
p.R1933X	c.5797C>T	14 (8)	13 (7)	9
p.D1941MfsX7	c.5818delG	5 (2)	5 (2)	9
p.E1945X	c.5833G>T	1 (1)	1 (1)	9
		75 (52)	67 (44)	

Abbreviations: Ref. = references.

### Liver studies in *MYH9*-RD patients

At the moment of the collection of data, the Italian Registry for *MYH9*-RD included 184 patients. The following data were extracted from the database: alanine aminotrasferase (ALT), aspartate aminotrasferase (AST) and gamma-glutamyltransferase (GGT). Elevation of liver enzymes was defined as a value above the upper normal limit (UNL) of the reference laboratories where the tests were carried out. The UNLs were established by the participant laboratories based on the 95^th^ percentile distribution of the serum concentrations of these enzymes in healthy blood donors in their respective geographic areas. In all patients medical history was recorded and physical examination was performed. Moreover, whenever available the results of liver ultrasound assessment and of additional biochemical tests were collected. These latter included: viral hepatitis markers (antibodies to HCV, HBV surface antigen, antibodies to HBV core antigen), antinuclear antibodies, anti-smooth muscle cell antibodies, anti-mitochondrial antibodies, anti-LKM and anti-microsomal antibodies, cupremia and plasma ceruloplasmin, serum α1-antitrypsin levels, serum ferritin and saturated ferritin, thyroid hormones, and anti-endomisium and anti-transglutaminase antibodies. All patients for which a known cause of elevation of liver enzymes was identified (e.g. infectious hepatitis, excessive alcohol intake, drug addiction, gallstone disease, metabolic syndrome, celiac disease, congestive heart failure, exposure to liver toxic agents) were excluded from the analysis.

Liver transient elastography (Fibroscan) was carried out in two patients and liver biopsy in one single case. Immunostaining for NMMHC-IIA was performed on paraffin-embedded liver sections by the PRB440P rabbit polyclonal antibody (Covance Research Products, Berkeley, CA, USA) diluted 1∶200 in PBS. The horseradish peroxidase LSAB 2 kit (Dako, Glostrup, Denmark) was used for secondary detection. The biopsy used as a control was an unaffected liver sample collected as part of the diagnostic procedures for clinical reasons unrelated to the present study and processed for staining after patient's informed consent.

### Liver studies in the control populations

The criteria for defining liver enzymes elevation in the three control populations were the same as for *MYH9*-RD patients. For the Ogliastra inhabitants, data of serum GGT were not available. For the participants to the Ogliastra survey, medical history was collected by a physician at the time of measurement of liver enzymes: subjects who referred a history of one of the known causes of liver damage listed above were excluded from the study.

### Statistical analysis

Continuous variables are reported as means ± standard deviation; categorical variables as total numbers and percentages. We explored the distribution of liver enzymes levels in the different patient populations examined. Possible associations between alterations of liver enzymes and the specific *MYH9* mutation, the residue of the NMMHC-IIA protein affected by mutation, the exon of *MYH9* involved by mutation, or the involved domain were investigated by Chi square test, after proper grouping of the *MYH9* mutations. Odds ratios (ORs) along with Chi square tests were used to assess differences in liver enzyme alterations between *MYH9*-RD patients and control populations, even excluding patients who underwent transfusion or receiving specific drugs to treat the clinical consequences of *MYH9* mutations. In addition, logistic regression was performed to take into account the potential confounding effect of age and sex.

## Results

Eighty-nine out of the 184 patients of the *MYH9*-RD registry had liver function tests recorded. Fourteen of them were excluded from the analysis because of the presence of an identifiable cause of liver damage (positive serology for HBV or HCV, alcohol or drug addiction, congestive heart failure).

Therefore, a total of 75 *MYH9*-RD patients belonging to 52 unrelated families were studied. Table 1 and 2 summarize their basic genotypic and clinical features. Liver enzymes data were compared with those of 7257 subjects from the Ogliastra population, 77 patients with ITP, and 32 patients with different forms of inherited thrombocytopenia not related to *MYH9* mutations. These included monoallelic Bernard-Soulier syndrome [19] (12 patients), autosomal-dominant thrombocytopenia deriving from *ANKRD26* mutations [20](11 patients), autosomal-dominant macrothrombocytopenia due to *ITGB3* mutation [21] (3 patients), and autosomal-dominant macrothrombocytopenia of undefined origin (6 patients) [22], [23]. Basic demographic features of the study populations are summarized in **Table 3**.

**Table 2:** Basic clinical features of the 75 *MYH9*-RD patients described in this study.

Gender - n° (%)		
Female	43	(57%)
Male	32	
Age - years		
Mean	31.3	
range	1–87	
Position of MYH9 mutation - n° (%)		
Motor domain	17	(23%)
Tail domain		58
Proteinuric nephropathy - n° (%)		
Yes	20	(27%)
No	55	
Sensorineural hearing loss - n° (%)		
Yes	40	(53%)
No	35	
Cataract - n° (%)		
Yes	10	(13%)
No	65	

Proteinuric nephropathy, sensorineural hearing loss, and cataracts were evaluated as previously reported (Pecci *et al*, Hum Mutat 2008, reference 2).

**Table 3:** Demographic characteristics of the study populations.

Population	N°	Men (%)	Age range [years]	Mean age (SD) [years]
***MYH9*** **-RD**	75	42.6	1–87	31.3 (19.9)
**Other inherited thrombocytopenias**	32	37.5	8–74	34.8 (19.4)
**ITP**	77	46.7	4–88	48.8 (24.8)
**Ogliastra inhabitants**	7257	45.4	3–105	43.2 (19.4)

### Liver test alterations in the *MYH9*-RD population

Thirty-eight out of 75 *MYH9*-RD patients (50.7%) presented an increase of AST or ALT in at least one determination (**Table 4**). ALT was the most frequently altered aminotransferase, since it was increased in 35 subjects (46.7%); twenty-nine of them (38.7% of total) had also a simultaneous increase of AST. Three patients (4%) showed an increase of only AST.

**Table 4:** Prevalence of liver enzyme alterations in the analyzed patient populations.

	ALT	AST	At least one (ALT,AST)	Both (ALT, AST)	GGT	At least one (ALT,AST,GGT)
***MYH9*** **-RD**	35/75 (46.7%)	32/75 (42.7%)	38/75 (50.7%)	29/75 (38.7%)	17/63 (27.0%)	39/69 (56.5%)
**Other inherited thrombocytopenias**	3/31 (9.7%)	1/30 (3.3%)	3/32 (9.4%)	1/29 (3.5%)	0/26 (0.0%)	3/26 11.5%)
**ITP**	5/77 (6.5%)	0/77 (0.0%)	5/77 (6.5%)	0/77 (0.0%)	4/71 (5.6%)	9/71 12.7%)
**Ogliastra inhabitants**	320/7257 (4.4%)	212/7257 (2.9%)	393/7257 (5.4%)	139/7257 (1.9%)	N.A.	N.A.

Data are presented as n° of patients with altered value/total n° of evaluable patients (%).

N.A. = not available.

Serum GGT was measured in 63 cases and found to be increased in 17 of them (27%). In particular, GGT was altered in 16 of the 32 evaluable subjects with an increase of aminotransferases (50%), while only one patient had isolated increase of GGT. The mean increase of aminotransferases was 1.9 (SD 0.7) fold the UNL for ALT and 1.4 (SD 0.4) for AST, while GGT were increased 2.7 fold (SD 1.6).

Among patients with alteration of liver enzymes, a total of 29 subjects had liver enzymes measured repeatedly (from 1 to 9 times, average 3.1, with intervals from 2 to 150 months, average 43.6 months) (**Table 5**). Out of these, 20 patients (69%) presented a stable picture of enzymatic alteration, with increased liver enzymes in all the measurements (mean number of repeated measurements, 2.9; mean interval, 34 months after the first measurement); four patients (17%) had a fluctuating enzymatic pattern, with normal findings at some of the intermediate measurements, while 5 patients (17%) experienced a normalization of the liver enzyme alterations after a mean observation time of 19.2 months. In none of the patients a clear worsening trend or a clinically relevant alteration of liver function was observed.

**Table 5:** Results of follow-up of 29 *MYH9*-RD patients with alterations of liver enzymes.

Liver enzyme	N° of patients with alteration at first measurement	N° of patients with alterations upon repeated measurements	N° of altered measurements/total n° of measurements	Mean interval between repeated measurements (range) [months]
**AST**	26	23 (88.4%)	86/115 (74.8%)	36.1 (2–150)
**ALT**	24	17 (70.8%)	58/111 (52.2%)	37.8 (2–150)
**GGT**	12	10 (88.3%)	34/78 (43.5%)	36.9 (5–120)

**Figure 1:**
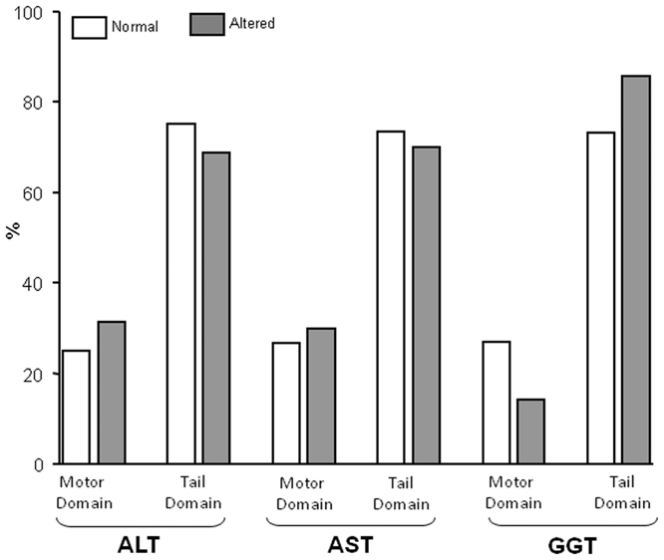
Frequency of abnormal liver tests depending on the location of the MYH9 gene mutation. Mutations involving the motor domain and tale domain regions of the gene are indicated. No significant differences in the distribution of liver test alteration between the two locations of the gene defect were evident.

Out of the 39 patients (belonging to 31 families) with alteration of liver enzymes, 56% were males and had a mean age of 30.5 years; 9 patients were 16 years-old or less. *MYH9-RD* patients with and without liver alterations did not differ significantly for gender, platelet count, and presence of other extra-hematological manifestations of *MYH9*-RD; moreover, no association was found between alterations of liver enzymes and patients' age (data not shown). There was no evident association between the elevation of liver enzymes (prevalence or degree of elevation) and the specific *MYH9* mutation, the residue of the NMMHC-IIA protein affected by mutation, or the involved domain (MD or TD, Figure 1). However, when causative mutations were grouped by the involved exon of *MYH9*, mutations of exon 25 (T1155I, T1155A, and R1162T) were associated with a higher prevalence of elevation of both aminotrasferases (p = 0.04) and with a higher level of increase of ALT (p = 0.02); a nearly significant association was observed also for a higher elevation of AST (p = 0.07). A possible cosegregation of mutations of exon 25 with altered liver enzymes, however, needs confirmation since only 4 patients with mutations involving this exon were analyzed.

### Prevalence of liver test alterations in the control populations

The prevalence of liver enzyme elevations in the Ogliastra population is reported in **Table 4**. A total of 393 out of 7257 subjects presented an alteration of ALT or AST in at least one determination (5.4%). The most frequent alteration was an increase of ALT (320 subjects, 4.4%), and 139 cases (1.9%) had a simultaneous increase of both aminotransferases. The remaining 73 subject (1.0%) showed an isolated elevation of AST. For subjects with liver enzymes above normal, the mean value was 1.7±0.7 fold the UNL for ALT and 1.5±0.7 for AST.

In the control group of patients with ITP, 5 out of 77 patients (6.5%) presented an alteration of ALT, while none of them had increased AST (**Table 4**). An isolated elevation of GGT was present in 4 of the 71 patients (5.6%) in which liver test was performed. For subjects with liver enzymes above normal, the average value was 1.34±0.1 fold the UNL for ALT, and 1.49±0.2 for GGT. When repeated enzyme measurements were carried out, only 1/20 patients (5%) presented an alteration in at least two determinations and none had a persistent alteration in all the determinations carried out.

In the control group of patients with *MYH9*-unrelated inherited thrombocytopenias, 3 out of 32 evaluable patients (9.4%) had an increase of at least one aminotransferase (**Table 4**). The most frequent alteration was an increase of ALT, which was present in three subjects (9.4%), one of which had also elevated AST, while none of the 26 evaluable patients had increased GGT. The mean increase was 1.48±0.12 fold the UNL for ALT and 2.18 for AST.

The OR to have an ALT elevation for *MYH9*-RD patients as compared to each of the three different control populations ranged from 8.2 to 18.9, to have an AST elevation from 21.6 to 24.7 and to have an elevation of at least one of ALT or AST from 8.0 to 14.8 (p<0.0003, at least) (**Table 6**). The OR to have an elevation of both aminotransferases ranged from 17.7 to 32.3 (p<0.0004, at least). When adjusted for age and sex the ORs to have a liver test alteration for *MYH9*-RD patients was even higher, ranging from 8.5 to 41.6 (not shown).

**Table 6:** Odds ratio to have aminotransferase elevations in *MYH9*-RD patients as compared with the three control populations.

OR vs.Enzyme alterations	ITP	p-value	Other inherited trombocyto-penias	p-value	Ogliastra inhabitants	p-value
**ALT**	12.6 (4.4–43.8)	0.00001	8.2 (2.2–44.8)	0.0003	18.9 (11.5–31.0)	0.00001
**AST**	N.A.^1^	-	21.6 (3.2–908.3)	0.0001	24.7 (14.8–40.8)	0.00001
**At least one (ALT or AST)**	14.8 (5.1–51.3)	0.00001	9.9 (2.7–54.2)	0.0001	8.0 (4.9–12.9)	0.00001
**Both (AST+ALT)**	N.A.^1^	-	17.7 (2.6–746.1)	0.0004	32.3 (18.9–54.0)	0.00001

Data represent ORs (95% CIs).

N.A. = not available

Note:

1 = Calculation of the OR is not possible since none of the 77 analysed ITP patients presented an elevation of AST.

**Figure 2:**
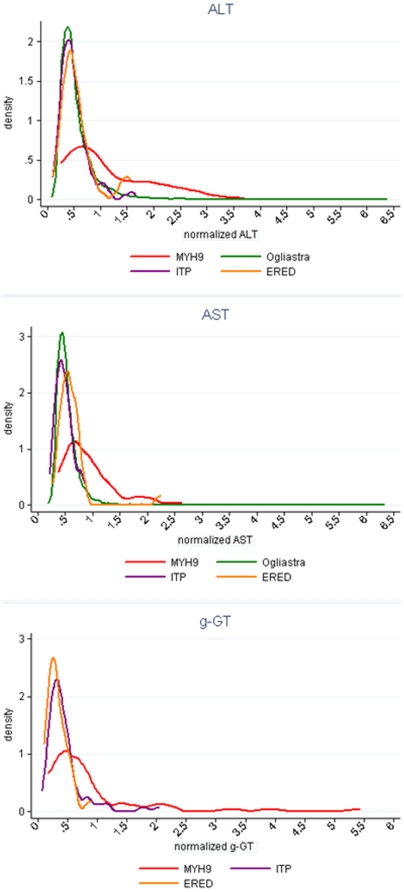
Distribution of liver test levels in the four populations studied. Data for ALT, AST and GGT distributions are shown.

The analysis of the distribution curve of liver enzyme levels in the different populations showed a markedly altered distribution in *MYH9*-RD patients, with a flat, tendentially bimodal distribution as compared with a rather typical Gaussian distribution in the ITP, inherited thrombocytopenias and Ogliastra populations (**Fig. 2**).

We also evaluated whether the increased prevalence of altered liver enzymes in *MYH9*-RD patients could be due to a higher prevalence of patients who underwent transfusion of blood products and therefore exposed to transfusion-related liver damage. In fact, 17 of the 75 investigated *MYH9*-RD patients (22.6%), 26 of 77 ITP patients (33.8%) and 4 of 32 subjects with other inherited thrombocytopenias (12.5%) had undergone platelet or red blood cell transfusions (a prevalence of transfused patients not significantly different between the three groups). Medical history about transfusions was not available for the Ogliastra population. The strongly increased prevalence and degree of elevation of liver enzymes in *MYH9*-RD patients with respect to each of the control populations was confirmed also when the analysis was restricted to the patients who had never undergone transfusions, even assuming that none of the subjects of the Ogliastra population received transfusions. Limiting the analysis to not transfused patients, the ORs to have a liver test alteration for *MYH9*-RD patients as compared with the control population ranged from 8.2 to 27.7 (p<0.001). Moreover, within the *MYH9*-RD population, no significant difference in the elevation of liver enzymes was observed between transfused and non-transfused patients (data not shown), demonstrating that previous transfusion did not exert a significant role in liver test alterations in our case series.

Finally, we considered the hypothesis that the increased frequency of markers of liver damage in *MYH9*-RD subjects could be secondary to the confounding effect of potentially hepatotoxic drugs administered to treat the clinical consequences of the *MYH9* mutations. In fact, 3 out of 75 *MYH9*-RD patients were under immunosuppressive treatment for a previous kidney transplantation and 8 were treated with ACE-inhibitors and/or angiotensin receptor blockers for proteinuria [24]. However, the strong association between *MYH9* mutations and alterations of liver enzymes was confirmed even when the analysis was restricted to patients not receiving any permanent drug treatment (data not shown).

### Additional liver studies in patients with *MYH9*-RD

Twenty-one out of 39 (53.8%) *MYH9*-RD patients with liver test abnormalities had a liver ultrasound examination carried out at least once. Of these 18 had normal findings and 3 presented a mild liver steatosis, defined as a “bright liver". Sometimes mildly enlarged liver, with fine tightly packed echoes that made the liver more echoic than the right kidney, was reported. Two patients had liver transient elastography (Fibroscan) carried out on one occasion: the median stiffness was within the normal range (F0).

Thirty-six of the 39 (92.3%) patients with altered liver tests had serum tests for B and C hepatitis: all of them were negative.

A total of 21 patients underwent the more detailed biochemical studies listed under methods: in all of them all these examinations resulted normal.

**Figure 3:**
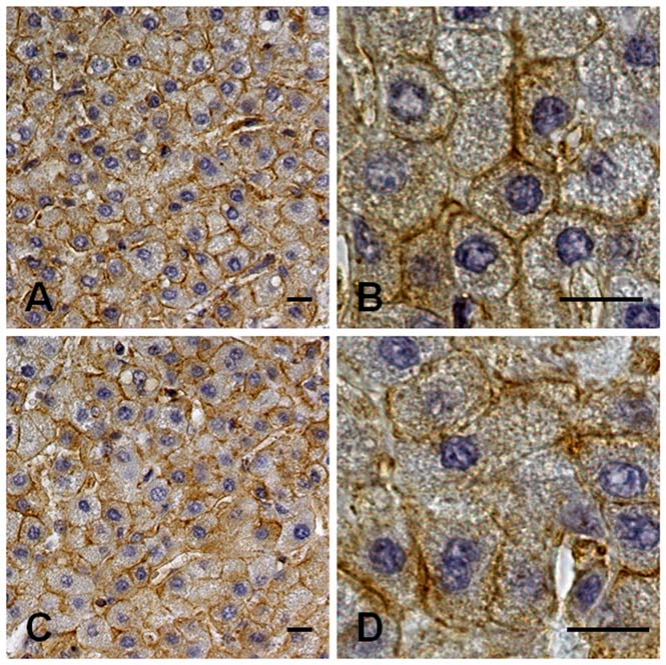
Immunohistochemistry for NMMHC-IIA in the liver biopsy from one patient with MYH9-RD. Liver biopsy from a 10-years-old patient with MYH9-RD caused by the p.T1155A mutation of MYH9 with persistently elevated AST, ALT and GGT. (A, B): Immunohistochemistry for NMMHC-IIA showed a signal (brown, horseradish peroxidase staining) concentrated close to the hepatocytes' plasma membrane. The distribution of NMMHC-IIA was not significantly different from that from a healthy control (C, D). Specimens were counterstained with Meyer's haematoxylin. Scale bars correspond to 10 µm.

In one patient, because of persistently elevated liver enzymes, a laparoscopic liver biopsy was performed after prophylactic transfusion of platelet concentrates. The patient was a 10-years-old boy with the p.T1155A MYH9 substitution and a 6-years history of persistently elevated ALT (59–303 U/L, UNL 38), AST (54–141 U/L, UNL 38), and GGT (136–151 U/L, UNL 50). Serum alkaline phosphatase was also slightly elevated, while bilirubin and cholinesterase were always within the normal range. He underwent all the examinations listed above, plus viral serology for HAV, CMV, EBV, cupruria, blood and urine aminoacid, all of which resulted normal. Histopathology revealed a preservation of hepatic architecture with scattered hepatocytes showing micro-macrovacuolar steatosis. Mild fibrosis was evident in the portal spaces and around the terminal veins. No inflammation, necrosis, apoptosis, regeneration or cholestasis were detectable, and the copper content was normal. Immunochemistry for NMMHC-IIA showed a positive reaction concentrated close to the hepatocytes' plasma membrane, particularly in the region of bile canaliculi. Cholangiocytes showed positivity mainly on the luminal side of plasma membrane. No significant differences in NMMHC-IIA distribution were detectable with respect to the liver biopsy used as a control (**Fig. 3**).

## Discussion

Our study in a large, well-characterized population of patients with *MYH9*-RD, highlighted a high prevalence of unexplained liver enzyme alterations, with over one half of patients showing abnormal serum enzyme levels suggestive of possible liver damage.

The prevalence of liver enzyme alterations in this population compares well or is even higher than that of the recognized non-congenital manifestations of the *MYH9*-RD syndrome, namely renal, hearing, and ocular defects, which are present in 16 to 60% of *MYH9*-RD patients [2], [6].

When compared with two different control populations of patients with thrombocytopenias from different causes, one with inherited thrombocytopenias other than *MYH9*-RD and the other with immune thrombocytopenia, or with a large population of people from a Sardinian geographic isolate, the ORs to have an elevation of liver enzymes for *MYH9*-RD patients ranged from 8.2 to 24.7, a highly significant increased risk. When adjusted for age and sex, the ORs were even higher, ranging from 8.5 to 41.6. No associated causes of liver enzyme alterations were detected, even upon more detailed liver studies, including repeated liver ultrasound or in two cases liver elastography and in one liver biopsy. Finally, a follow-up ranging from 2 to 150 months (mean 44) showed no trend towards a worsening of the observed liver test alterations.

These data indicate that an elevation of liver enzymes is a feature of the clinical spectrum of *MYH9*-RD, but that this alterations seem benign, with no significant evolution towards clinically relevant liver damage or dysfunction. Indeed, even in *MYH9*-RD patients of an advanced age, in which an elevation of liver enzymes was detected since the first diagnosis of their disease, there was not one single case developing liver failure. Differently from what happens with the other clinical features of the disease which are more severe in patients carrying *MYH9* mutations affecting the MD of the protein, no clear genotype/phenotype correlations were evident for the elevation of liver enzymes; however, a possible cosegregation of the mutations involving exon 25 with altered liver enzymes seems to emerge but this needs confirmation since only 4 patients with mutations involving this exon were analyzed.

Our study has some potential limitations: *MYH9*-RD patients may undergo platelet or blood transfusions more frequently than healthy controls during their lifetime, and therefore they may more prone to transfusion-related infectious liver injury. However, the strongly increased prevalence of altered liver enzymes in *MYH9*-RD patients was confirmed even when the analysis was restricted to patients that had never received platelet or blood transfusions. Moreover, the vast majority of our *MYH9*-RD patients (92.3%) had been tested for B and C viral hepatitis, and all those found positive were excluded from the analysis. In addition, even assuming that the three subjects that had not been tested were all HBV or HCV positive, the proportion of *MYH9*-RD patients with unexplained liver enzyme elevation remains significantly higher than that of controls (data not shown). Finally, the possible confounding effect of potentially hepatotoxic drugs administered to treat the clinical consequences of *MYH9* mutations could reasonably be ruled out. Another limitation is that a laboratory follow-up was available for only a fraction of the *MYH9*-RD population and that follow-up was not of a sufficient duration (maximum 150 months) to exclude a possible late worsening of liver function. However, the observations that in a large case-series of *MYH9*-RD patients, including cases aged 80 or more, not one single case evolved into liver failure/cirrhosis and that imaging studies never showed significant liver structural alterations suggest that liver test alterations in this genetic syndrome do not lead to liver function impairment. This differs from kidney involvement in *MYH9*-RD, which, when occurs, tends to evolve progressively into end-stage renal failure [2], [6]. Of course a definitive conclusion on the benign course of liver involvement in MYH9-RD requires confirmatory studies in large case series with longer follow-up.

In the hepatocytes non-muscle myosin of class II has well-recognized functions correlated with bile secretion. Myosin II was found to be enriched in the actin microfilament network around the bile canaliculus and was identified as the essential motor for the bile canalicular contraction [25]–[27]. Moreover, myosin II is involved in vesicle trafficking between the cytoplasmic compartment and plasma membrane and regulates the apical membrane expression of several transporters associated with bile secretion, such as the bile salt export pump, whose genetic defects are associated with some forms of familiar intrahepatic cholestasis [28], [29]. Recent studies identified additional key roles for myosin II in hepatocytes, since it was implicated in postnatal hepatocytes polyploidization [30] spatial reorganization of hepatocytes during development and liver regeneration [31], and cell cycle progression and motility [32], [33]. Although we have no data to provide a mechanistic explanation of liver enzyme alterations in *MYH9*-RD, the observation that the myosin IIA loss-of-function of *MYH9*-RD patients primarily results in a phenotype consistent with hepatocellular damage rather than a cholestatic disease seems more consistent with the functions of myosin in hepatocyte polyploidization, regeneration, cell cycle and motility [30]–[33].

In conclusion, our study shows that an alteration of liver enzymes is a feature of the *MYH9*-RD syndrome, a defect however that does not appear to have a poor prognostic value.
